# Evaluation of using ICD‐10 code data for respiratory syncytial virus surveillance

**DOI:** 10.1111/irv.12665

**Published:** 2019-06-17

**Authors:** Wei Cai, Kristin Tolksdorf, Siddhivinayak Hirve, Ekkehard Schuler, Wenqing Zhang, Walter Haas, Silke Buda

**Affiliations:** ^1^ Respiratory Infections Unit, Department for Infectious Disease Epidemiology Robert Koch Institute Berlin Germany; ^2^ Global Influenza Programme World Health Organization Geneva Switzerland; ^3^ HELIOS KLINIKEN GmbH Berlin Germany

**Keywords:** epidemiology, ICD‐10 code, respiratory syncytial virus, sensitivity, specificity, surveillance

## Abstract

**Background:**

Respiratory syncytial virus (RSV) is the most common cause of acute lower respiratory tract infection (ALRI) in young children. ICD‐10‐based syndromic surveillance can transmit data rapidly in a standardized way.

**Objectives:**

We investigated the use of RSV‐specific ICD‐10 codes for RSV surveillance.

**Methods:**

We performed a retrospective descriptive data analysis based on existing ICD‐10‐based surveillance systems for ALRI in primary and secondary care and a linked virological surveillance in Germany. We described RSV epidemiology and compared the epidemiological findings based on ICD‐10 and virological data. We calculated sensitivity and specificity of RSV‐specific ICD‐10 codes and in combination with ICD‐10 codes for acute respiratory infections (ARI) for the identification of laboratory‐confirmed RSV infections.

**Results:**

Based on the ICD‐10 and virological data, epidemiology of RSV was described, and common findings were found. The RSV‐specific ICD‐10 codes had poor sensitivity 6% (95%‐CI: 3%‐12%) and high specificity 99.8% (95%‐CI: 99.6%‐99.9%). In children <5 years and in RSV seasons, the sensitivities of RSV‐specific ICD‐10 codes combined with general ALRI ICD‐10 codes J18.‐, J20.‐ and with J12.‐, J18.‐, J20.‐, J21.‐, J22 were moderate (44%, 95%‐CI: 30%‐59%). The specificities of both combinations remained high (91%, 95%‐CI: 86%‐94%; 90%, 95%‐CI: 85%‐94%).

**Conclusions:**

The use of RSV‐specific ICD‐10 codes may be a useful indicator to describe RSV epidemiology. However, RSV‐specific ICD‐10 codes underestimate the number of actual RSV infections. This can be overcome by combining RSV‐specific and general ALRI ICD‐10 codes. Further investigations are required to validate this approach in other settings.

## 1 INTRODUCTION

Respiratory syncytial virus (RSV) is a worldwide distributed pathogen of acute respiratory infection (ARI) of all ages. In infants and young children, RSV is the most common cause of acute lower respiratory tract infection (ALRI) and a major cause of hospital admission for ALRI. Worldwide in 2015, 21.6‐50.3 million RSV‐associated ALRI episodes occurred in children younger than 5 years, with about 2.7‐3.8 million hospital admissions.^1^, ^2^

Currently, only passive immunization with palivizumab against RSV is available for children at high risk.^3^ In 2015, the World Health Organization (WHO) Product Development for Vaccines Advisory Committee highlighted the development of safe and efficacious RSV vaccines for global use. Several novel RSV vaccines have shown promising results in clinical trials and are expected to enter the market by 2025.^4^, ^5^ The planning of future RSV vaccination strategies and the evaluation of RSV vaccination impact rely on timely RSV epidemiological data and long‐term observation of RSV seasonality through RSV surveillance systems.

International Statistical Classification of Diseases and Related Health Problems (ICD) diagnosis codes have been used to describe the burden of respiratory diseases and the impact of vaccination.^6^, ^7^, ^8^, ^9^ ICD‐based digital syndromic surveillance is a relatively novel surveillance practice, compared to the traditional surveillance. It can not only describe epidemiology of disease, but also capture and transmit data rapidly in a standardized and sustainable way at lower costs, and provide very early warning of potential public health threats.^10^, ^11^, ^12^

The Robert Koch Institute (RKI) established the 10th revision of ICD (ICD‐10)‐based digital syndromic surveillance systems for influenza and other ARI in primary and secondary care in Germany (Appendix S1). In primary care, general practitioners, internists, and pediatricians of sentinel practices report influenza and other ARI data voluntarily through a syndromic influenza surveillance system. This system has been linked with a virological surveillance and a sentinel electronic data collection system based on ICD‐10 codes (SEED^ARE^).^13^ SEED^ARE^ was evaluated as a valid system for syndromic influenza surveillance.^14^ In secondary care, an ICD‐10 code‐based surveillance system for severe acute respiratory infections (ICOSARI) has been implemented in cooperation with a private hospital network in Germany.^15^

Studies estimating validity of ICD diagnosis codes for the identification of laboratory‐confirmed influenza have shown mixed results.^14^, ^16^, ^17^, ^18^ So far, few studies have looked at accuracy of RSV‐specific ICD‐10 diagnosis codes for the identification of true RSV infections. To our knowledge, only Pisesky et al^19^ reported high sensitivity (97.9%, 95%‐CI: 95.5%‐99.2%) and specificity (99.6%, 95%‐CI: 98.2%‐99.8%) of RSV‐specific ICD‐10 codes for the identification of hospitalized RSV among children.

The aim of this study was to evaluate the use of RSV‐specific ICD‐10 diagnosis codes for RSV surveillance.

## 2 METHODS

We performed a retrospective descriptive data analysis based on the data derived from ICD‐10‐based influenza and other ARI surveillance systems SEED^ARE^ and ICOSARI, and from the virological surveillance at the RKI. The SEED^ARE^ system has functioned since 2007, the virological surveillance since 2010, and ICOSARI since 2015. The datasets of ICOSARI for the years 2009 to 2014 were collected retrospectively. The Appendix S1 provides details on the surveillance participants, data collection methods, collected data, total number of collected data, and study period (13, 14, 15, Appendix S1).

The SEED^ARE^ system was approved by the German Federal Commissioner for Data Protection and Freedom of Information, and the ICOSARI system by the RKI and HELIOS Kliniken GmbH data protection authority. As SEED^ARE^ and ICOSARI involved no interventions and the analysis was based on anonymized data only, no ethical clearance was required for them.^14^, ^15^ The virological surveillance activities were approved by the German Federal Commissioner for Data Protection and Freedom of Information and the Ethical Committee of the Charité, Universitätsmedizin, Berlin.

We defined a RSV‐ICD‐case based on SEED^ARE^ data as a medical consultation with any of the three RSV‐specific ICD‐10 code diagnoses (J12.1 RSV pneumonia, J20.5 acute bronchitis due to RSV, and J21.0 acute bronchiolitis due to RSV).^6^ We defined a RSV‐ICD‐case based on ICOSARI data as a hospitalization with any of the three RSV‐specific ICD‐10 code diagnoses as primary discharge diagnosis. In the virological surveillance, we defined a confirmed‐RSV‐case as a by real‐time reverse transcriptase polymerase chain reaction (rtRT‐PCR) confirmed RSV sample. In each data source, a RSV season was defined as the weeks when cumulative number of RSV‐ICD‐cases or confirmed‐RSV‐cases exceeded 1.2% of total RSV‐ICD‐cases or confirmed‐RSV‐cases. One gap week below the threshold was allowed.^20^, ^21^

We estimated number of RSV‐ICD‐cases and confirmed‐RSV‐cases by gender, age group (0‐1, 2‐4, 5‐14, 15‐34, 35‐49, 50‐59, ≥60 years), and calendar week based on each data source, respectively.

We identified the sentinel practices that participated in both SEED^ARE^ and the virological surveillance concurrently by practice‐ID. We matched the medical consultations of SEED^ARE^ with virological samples by practice‐ID, age, gender, consultation date, and sampling date. Only one‐to‐one matches were included for the further data evaluation. We calculated sensitivity of RSV‐specific ICD‐10 code diagnosis as proportion of RSV‐ICD‐cases among confirmed‐RSV‐cases, and specificity as proportion of non‐RSV‐ICD‐cases among non‐confirmed‐RSV‐cases of the identified practices. We calculated sensitivity and specificity of RSV‐specific ICD‐10 code diagnosis among young children, in RSV seasons, and combined with different general ARI ICD‐10 codes J06.‐ acute upper respiratory infections of multiple and unspecified sites (J06, J06.0, J06.8, J06.9), J11.‐ influenza, virus not identified (J11, J11.0, J11.1, J11.8), J12.‐ viral pneumonia, not elsewhere classified (J12, J12.8, J12.9), J18.‐ pneumonia, organism unspecified (J18, J18.0, J18.8, J18.9), J20.‐ acute bronchitis (J20, J20.8, J20.9), J21.‐ acute bronchiolitis (J21, J21.8, J21.9), J22 unspecified ALRI, and B34.9 unspecified viral infection, respectively.^6^ The sensitivities and specificities were calculated with 95% confidence interval (95%‐CI). Additionally, we compared RSV‐ICD‐cases with confirmed‐RSV‐cases of the identified practices by calendar week.

We used Stata (version 15) and microsoft excel 2010 for the data analysis.

## 3 RESULTS

### 3.1 Primary care

#### 3.1.1 Descriptive analysis of RSV‐ICD‐cases based on SEED^ARE^ data

A total of 1165 RSV‐ICD‐cases were identified from the SEED^ARE^ database from week 40/2007‐13/2017. Among those, 338 (29%) were diagnosed with J12.1, 432 (37%) with J20.5, and 395 (34%) with J21.0. The proportion of RSV‐ICD‐cases among all ARI‐ICD‐cases was 0.1%.

**Figure 1:**
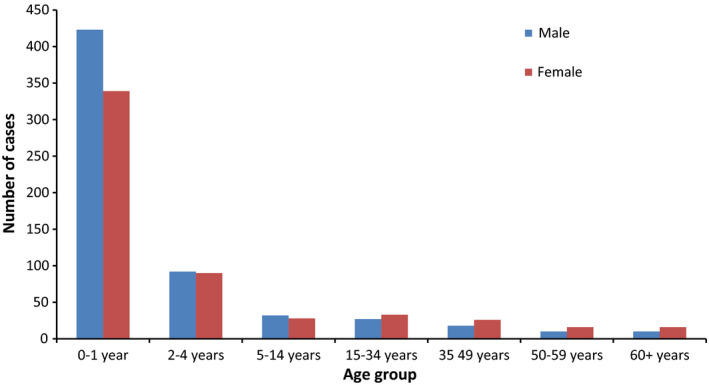
Number of RSV‐ICD‐cases by age group and gender based on SEED^ARE^, week 40/2007‐13/2017

About two‐thirds (765; 66%) of RSV‐ICD‐cases were children aged <2 years. The number of RSV‐ICD‐cases declined rapidly from 2 years of age and remained at a constantly low level from 5 years of age onwards. Under 2 years of age, the number of RSV‐ICD‐cases was higher in boys (423) than in girls (339; Figure 1).

The cumulative number (88) of the RSV‐ICD‐cases within the observed 10‐year period peaked in the 8th calendar week, and the proportion (0.3%) of RSV‐ICD‐cases among all ARI‐ICD‐cases peaked in the 2nd calendar week. The RSV season on average was from 41st to 16th calendar week with the season length of 28 weeks. Within the RSV seasons, 92% (1075) RSV‐ICD‐cases were captured.

#### 3.1.2 Descriptive analysis of confirmed‐RSV‐cases based on virological surveillance data

From week 40/2010‐18/2017, 1785 (8%) respiratory specimens of ARI or influenza‐like illness (ILI) patients were RSV positive.

**Figure 2:**
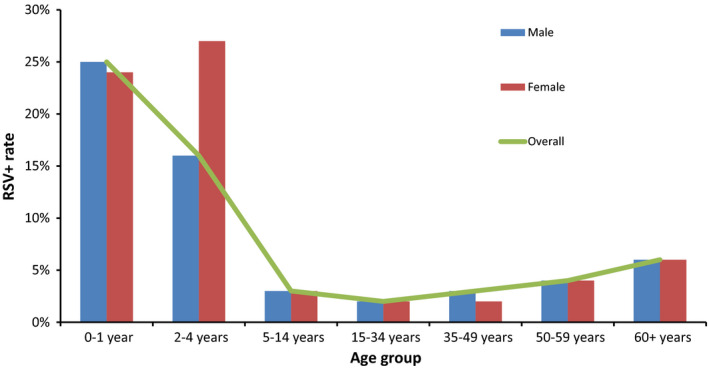
RSV‐positive rate by age group and gender based on virological surveillance, week 40/2010‐18/2017

The highest RSV positive rate (25%; 659) was among children aged <2 years. The RSV positive rate decreased from 2 years of age, reached the lowest level in the age group 15‐34 years (2%; 98), then increased slightly again, and reached 6% (145) at the age of 60 years and older. Under 2 years of age, the RSV‐positive rate was higher among boys (25%; 378) than girls (24%; 270; Figure 2).

The cumulative number (143) of confirmed‐RSV‐cases peaked in the 6th calendar week, and the RSV positive rate (18%) peaked in the 52nd calendar week. The RSV season on average was from 48th to 15th calendar week with the season length of 20 weeks. Within the RSV seasons, 94% (1671) confirmed‐RSV‐cases were captured.

### 3.2 Integration of RSV data of practices participated in SEED^ARE^ and virological surveillance

**Figure 3:**
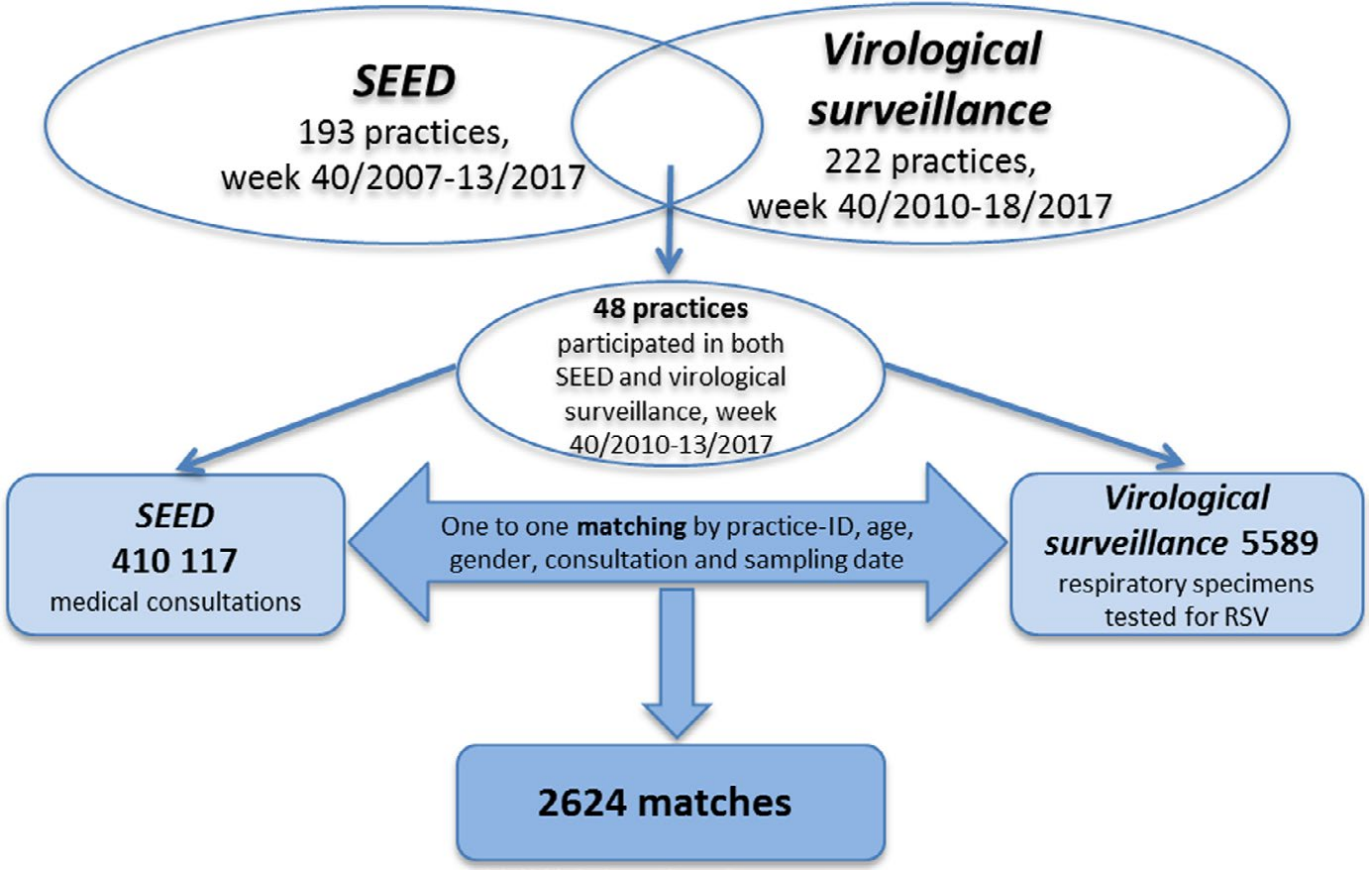
Integration of RSV data of practices participated in both SEED^ARE^ and virological surveillance, week 40/2010‐13/2017

Forty‐eight sentinel practices participated in both SEED^ARE ^and the virological surveillance from week 40/2010‐13/2017. In total, 5589 respiratory specimens of the 48 practices were tested for RSV. Of those, 400 (7%) were RSV positive, and 2624 (47%) could be matched with the medical consultations based on SEED^ARE^ one to one (Figure 3).

Overall, the sensitivity of RSV‐specific ICD‐10 code diagnosis was 6% (95%‐CI: 3%‐12%), and the specificity was 99.8% (95%‐CI: 99.6%‐99.9%). The sensitivity (16%, 95%‐CI: 7%‐29%) increased among children aged <5 years and during the RSV seasons based on the virological data (48th–15th calendar week), and the specificity (99.5%, 95‐CI: 97.5%‐99.9%) remained high. In children aged <5 years and in RSV seasons, the sensitivities of RSV‐specific ICD‐10 codes combined with general ALRI ICD‐10 codes J18.‐, J20.‐, and with J12.‐, J18.‐, J20.‐, J21.‐, J22 both reached 44% (95%‐CI: 30%‐59%), and the specificities of the two combinations were still at a high level (91%, 95%‐CI: 86%‐94%; 90%, 95%‐CI: 85%‐94%). The sensitivity of RSV‐specific ICD‐10 codes combined with all general ARI ICD‐10 codes was 90% (95%‐CI: 78%‐97%), whereas the specificity was 16% (95%‐CI: 11%‐21%; Table 1).

**Table 1:** Sensitivities and specificities of RSV‐specific ICD‐10 code diagnosis combined with different general ARI ICD‐10 codes of the practices participated in both SEED^ARE^ and virological surveillance, week 40/2010‐13/2017

	Sensitivity		Specificity	
	%	95%‐CI	%	95%‐CI
RSV codes^a^	6	3‐12	99.8	99.6‐99.9
**<2 y of age**				
RSV codes	8	2‐22	99.4	95.6‐99.9
**<5 y of age**				
RSV codes	14	6‐26	99.6	98‐99.9
**In RSV seasons** b				
RSV codes	7	3‐12	99.8	99.5‐99.9
**<5 y of age and in RSV seasons**				
RSV codes	16	7‐29	99.5	98‐99.5
RSV codes + J06.‐c	48	34‐63	62	55‐68
RSV codes + J11.‐d	30	18‐45	75	68‐80
RSV codes + J12.‐e	16	7‐29	99.5	98‐99.9
RSV codes + J18.‐f	30	18‐45	98	95‐99
RSV codes + J20.‐g	30	18‐45	92	88‐95
RSV codes + J21.‐h	16	7‐29	99.5	98‐99.9
RSV codes + J22	16	7‐29	99	97‐99.9
RSV codes + B34.9	28	16‐42	80	74‐85
RSV codes + J18.‐, J20.‐	44	30‐59	91	86‐94
RSV codes + J18.‐, J20.‐, B34.9	56	41‐70	72	65‐77
RSV codes + J11.‐, J18.‐, J20.‐, B34.9	62	47‐75	48	42‐55
RSV codes + J12.‐, J18.‐, J20.‐, J21.‐, J22	44	30‐59	90	85‐94
RSV codes + all general ARI codes^i^	90	78‐97	16	11‐21

a RSV codes: RSV‐specific ICD‐10 codes J12.1, J20.5, J21.0.

b RSV season: 48th‐15th calendar week.

c J06.‐: J06, J06.0, J06.8, J06.9.

d J11.‐: J11, J11.0, J11.1, J11.8.

e J12.‐: J12, J12.8, J12.9.

f J18.‐: J18, J18.0, J18.8, J18.9.

g J20.‐: J20, J20.8, J20.9.

h J21.‐: J21, J21.8, J21.9.

i All general ARI codes: J06, J06.0, J06.8, J06.9, J11, J11.0, J11.1, J11.8, J12, J12.8, J12.9, J18, J18.0, J18.8, J18.9, J20, J20.8, J20.9, J21, J21.8, J21.9, J22, B34.9.

**Figure 4:**
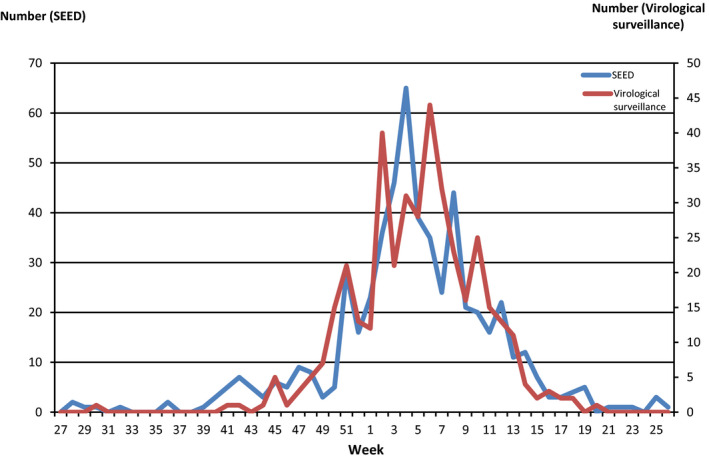
Cumulative number of RSV‐ICD‐cases based on SEED^ARE^ and cumulative number of confirmed‐RSV‐cases based on virological surveillance by calendar week in the practices participated in both SEED^ARE^ and virological surveillance, week 40/2010‐18/2017

**Figure 5:**
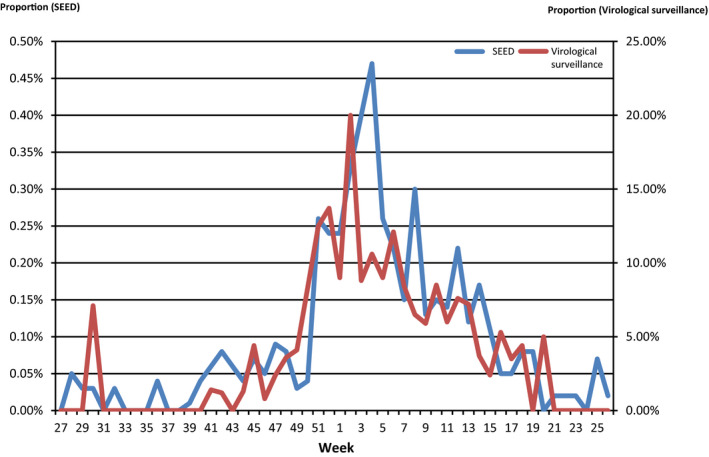
Proportion of RSV‐ICD‐cases based on SEED^ARE^ and RSV positive rate based on virological surveillance by calendar week in the practices participated in both SEED^ARE^ and virological surveillance, week 40/2010‐18/2017

Figures 4 and 5 indicate number and proportion of RSV‐ICD‐cases based on the SEED^ARE^ and confirmed‐RSV‐cases based on the virological surveillance in the 48 practices by calendar week, respectively. The trends of the curves were similar.

### 3.3 Secondary care

#### 3.3.1 Descriptive analysis of RSV‐ICD‐cases based on ICOSARI data

Among 1 417 700 respiratory disease, hospitalizations from week 01/2009‐15/2017, 7345 (0.5%) were hospitalizations with any of the RSV‐specific ICD‐10 codes as primary or secondary discharge diagnosis, and 3154 (0.2%) as admission diagnosis. Of the 7345 RSV hospitalizations, 6918 (94%) were with RSV‐specific ICD‐10 codes as primary discharge diagnosis. Of the three RSV‐specific ICD‐10 codes, J21.0 was most frequently diagnosed as primary discharge (2705; 39%) and also admission diagnosis (1679; 53%).

**Figure 6:**
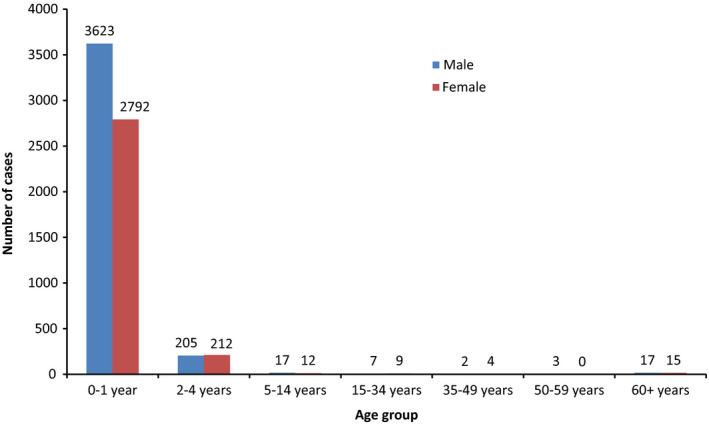
Number on RSV‐ICD‐cases by age group and gender based on ICOSARI, week 01/2009‐15/2017

Of the 6918 RSV‐ICD‐cases, 93% (6415) were children aged <2 years. The number of RSV‐ICD‐cases declined rapidly from 2 years of age. From 5 years of age, only a few RSV‐ICD‐cases were identified in each age group. In the age group 60 years and older, the number (32) of RSV‐ICD‐cases rose slightly. Under 2 years of age, number of RSV‐ICD‐cases was higher among boys (3623) than girls (2792; Figure 6).

The cumulative number (535) of RSV‐ICD‐cases peaked in the 5th calendar week, and the proportion (2.1%) peaked in the 52nd calendar week. The RSV season on average was from 48th to 16th calendar week with the season length of 21 weeks. Within the RSV seasons, 93% (6444) RSV‐ICD‐cases were captured.

## 4 DISCUSSION

Using ICD‐10‐based surveillance, we identified age groups under high risk of RSV, and successfully described general trends and seasonality of RSV in primary and secondary care in Germany, as confirmed by data from the virological surveillance system. In primary care, RSV‐specific ICD‐10 codes had poor sensitivity and high specificity for the identification of laboratory‐confirmed RSV infections. In young children, two combinations of RSV‐specific ICD‐10 codes with general ALRI ICD‐10 codes increased the sensitivity without decreasing the specificity much.

The described RSV epidemiology based on ICD‐10 code and virological data showed many common findings. Especially, high number of RSV cases among young children, and higher number of RSV cases among young boys than young girls were found in ICD‐10 and also in virological data sources. These findings are also in accordance with those reported in the literature.^1^, ^2^, ^22^, ^23^

In the present study, the proportion of young children among all RSV‐ICD‐cases was higher in secondary care based on ICOSARI than in primary care based on SEED^ARE ^data. This is in agreement with the clinical observation that RSV infection is normally more serious in young children and is a major cause of hospital admission in this group.^1^, ^2^ Bronchiolitis is a very severe manifestation of RSV disease mainly affecting young children, whereas bronchitis is more common in older children and adults.^24^, ^25^ Of the three RSV‐specific ICD‐10 codes, J21.0 (acute bronchiolitis due to RSV) was most frequently diagnosed in secondary care based on ICOSARI and J20.5 (acute bronchitis due to RSV) in primary care based on SEED^ARE^.

Based on the three data sources, the RSV season onset ranged from mid‐October to end‐November, the season offset was in mid‐April, and the peak of season ranged from end‐January to mid‐February in Germany. The RSV season length ranged from 20 to 28 weeks. The RSV seasons captured most of the RSV cases. RSV season onset, offset, peak week, and season length based on ICOSARI and virological surveillance were similar. Based on SEED^ARE^ outpatient surveillance, the season began earlier. The outpatient syndromic surveillance may provide earlier warning of RSV spread compared to the ICOSARI inpatient syndromic surveillance and the traditional virological surveillance. The RSV seasonality based on present study correlates well with the literature that the peak of RSV season is in winter months in Germany and areas with similar climate in the northern hemisphere.^26^, ^27^, ^28^ The median length of RSV seasons in the present study was longer than the median length of RSV seasons in the 15 European countries.^21^

The similar RSV seasonality based on ICD‐10 data in secondary care and virological data in primary care, and the similar RSV trends based on ICD‐10 and virological data of the practices participated in both SEED^ARE^ and virological surveillance indicated that the RSV‐specific ICD‐10 code data reflected the true temporal distribution of RSV infection.

We found that RSV‐specific ICD‐10 codes were less sensitive and highly specific for the identification of laboratory‐confirmed RSV infections in primary care. Low sensitivity of the ICD‐10 codes was also reported for influenza.^16^, ^17^, ^18^ In Germany, laboratory diagnostic tests are not always performed for suspected RSV infections in primary care. Even if testing is performed, an ICD‐10 code diagnosis will probably no longer be recoded when laboratory findings are only available in the practice a few days later after the medical consultation. Therefore, suspected and also laboratory‐confirmed RSV infections may be encoded with general ARI ICD‐10 codes. These could be the reasons why most of the laboratory‐confirmed RSV cases were not encoded with RSV‐specific ICD‐10 codes in the sentinel practices which participated in both the SEED^ARE^ and virological surveillance in the present study. In preparation for the present study, the RKI performed a survey to explore RSV coding behavior in primary care in Germany. The results of the survey are in line with the explanations above (unpublished data).

In children aged <5 years and in RSV seasons, the sensitivity of RSV‐specific ICD‐10 codes grew more than twofold, and the specificity remained high. Physicians were probably more likely to encode with RSV‐specific ICD‐10 codes for young children and in RSV seasons since RSV is more common in this group and during this time period. In the present study, we tried estimating the sensitivities and specificities of RSV‐specific ICD‐10 codes combined with different general ARI ICD‐10 codes. RSV‐specific ICD‐10 codes combined with two groups of general ALRI ICD‐10 codes achieved moderate sensitives and high specificities. The high sensitivity of RSV‐specific ICD‐10 codes combined with all general ARI ICD‐10 codes suggests that in addition to RSV‐specific ICD‐10 codes, most laboratory‐confirmed RSV infections were diagnosed with general ARI ICD‐10 codes. Thus, the misclassification related to inaccurate labeling of RSV infections with other disease‐ or pathogen‐specific ICD‐10 codes was uncommon in the present study.

The present study has some limitations. The sensitivity and specificity of RSV‐specific ICD‐10 code diagnoses in secondary care could not be evaluated on a case by case basis since virological data of the ICOSARI network were not available for the present study. However, in the ICOSARI network, suspected RSV cases in young children were tested by rapid antigen detection tests and rtRT‐PCR, and laboratory‐confirmed RSV infections were encoded with RSV‐specific ICD‐10 codes. Although whether the testing and coding took place in a 100% frequency is not verified, these have been as a standard procedure in the pediatric units and the coding quality could have increased in recent years (personal communication). In addition, high validity has been reported in the literature for RSV‐specific ICD‐10 codes for the identification of hospitalized RSV among children.^19^

The RSV coding behavior of physicians in primary care may vary during and out of RSV season, based on use of laboratory diagnostics, age of patient, and level of coding awareness. The differences in coding behavior may lead to information bias. The number of confirmed‐RSV‐cases and RSV‐ICD‐cases increased slightly among older adults based on virological as well as ICOSARI data, and it remained at a low level based on SEED^ARE^. The RSV infection normally goes unrecognized with milder symptoms among adults; however, it is a common pathogen of ARI in older adults and can lead to severe disease.^29^, ^30^ Therefore, the RSV infections were probably underestimated among older adults in SEED^ARE^. This could be another limitation. However, the evaluation of the accuracy of ICD‐10 codes was exactly the objective of the present study due to the potential information bias.

The present study was based on anonymized data. According to practice‐ID, age, gender, consultation date, and sampling date alone, more than half of the virological samples could not be matched to medical consultations one to one and were excluded for the evaluation of sensitivity and specificity of RSV‐specific ICD‐10 codes which might lead to selection bias. However, the probability of the selection bias was low since no conspicuous deviations were found between the matched and the excluded virological data (data not shown).

## 5 CONCLUSIONS

The use of RSV‐specific ICD‐10 code data may be a useful indicator to identify age groups under high risk of RSV, to monitor general trends, and to observe seasonality of RSV. The RSV epidemiology based on ICD‐10 code data from different data sources and virological data showed similar age and sex distribution, percent positivity, and seasonality patterns. Therefore, RSV‐specific ICD‐10 codes are appropriate for RSV surveillance. However, in primary care, RSV‐specific ICD‐10 code diagnosis was less sensitive, and relying on RSV‐specific ICD‐10 codes alone will underestimate the actual number of RSV infections. RSV‐specific ICD‐10 codes combined with the general ALRI ICD‐10 codes J18.‐, J20‐., and with J12.‐, J18.‐, J20.‐, J21.‐, J22 achieved moderate sensitives and high specificities, respectively. Thus, when establishing an ICD‐10‐based digital RSV surveillance system in young children, an extended ICD‐10‐based RSV case definition using the two combinations of ICD‐10 codes seems to better capture the true RSV disease burden. Further investigations are required to validate the use of the two combinations of ICD‐10 codes in RSV surveillance systems in other countries as the RSV coding behavior may differ in different countries, to find out an even better combination of ICD‐10 codes for the identification of RSV infections in primary care, and to evaluate the use of RSV‐specific ICD‐10 codes in secondary care.

## CONFLICT OF INTEREST

None.

## AUTHOR CONTRIBUTIONS

Wei Cai, Silke Buda, Walter Haas, Siddhivinayak Hirve, and Wenqing Zhang were involved in designing the study. Kristin Tolksdorf, Silke Buda, and Ekkehard Schuler participated in the collection of ICOSARI data. Wei Cai analyzed the data, and Kristin Tolksdorf helped analyze the data. Wei Cai drafted the manuscript. All authors reviewed and approved the final manuscript.

## References

[1] Shi T , McAllister DA , O'Brien KL , et al. Global, regional, and national disease burden estimates of acute lower respiratory infections due to respiratory syncytial virus in young children in 2015: a systematic review and modelling study. Lancet. 2017;390(10098):946‐958. 28689664 10.1016/S0140-6736(17)30938-8 PMC5592248

[2] Hall CB , Weinberg GA , Iwane MK , et al. The burden of respiratory syncytial virus infection in young children. N Engl J Med. 2009;360(6):588‐598. 19196675 10.1056/NEJMoa0804877 PMC4829966

[3] Taleb SA , Al Thani AA , Al Ansari K , Yassine HM . Human respiratory syncytial virus: pathogenesis, immune responses, and current vaccine approaches. Eur J Clin Microbiol Infect Dis. 2018;37(10):1817‐1827. 29876771 10.1007/s10096-018-3289-4

[4] Modjarrad K , Giersing B , Kaslow DC , Smith PG , Moorthy VS ; WHO RSV Vaccine Consultation Expert Group . WHO consultation on respiratory syncytial virus vaccine development report from a world health organization meeting held on 23–24 March 2015. Vaccine. 2016;34(2):190‐197. 26100926 10.1016/j.vaccine.2015.05.093 PMC6858870

[5] Mazur NI , Higgins D , Nunes MC ; Respiratory Syncytial Virus Network (ReSViNET) Foundation . The respiratory syncytial virus vaccine landscape: lessons from the graveyard and promising candidates. Lancet Infect Dis. 2018;S1473‐3099(18):30292‐30295. 10.1016/S1473-3099(18)30292-5 29914800

[6] World Health Organization . International statistical classification of diseases and related health problems 10th revision. ICD‐10 Online version: 2016. http://apps.who.int/classifications/icd10/browse/2016/en

[7] D'Onise K , Raupach JC . The burden of influenza in healthy children in South Australia. Med J Aust. 2008;188(9):510‐513. 18459921 10.5694/j.1326-5377.2008.tb01763.x

[8] Bonacruz‐Kazzi G , McIntyre P , Hanlon M , Menzies R . Diagnostic testing and discharge coding for whooping cough in a children's hospital. J Paediatr Child Health. 2003;39(8):586‐590. 14629523 10.1046/j.1440-1754.2003.00244.x

[9] Moore HC , de Klerk N , Richmond P , Lehmann D . A retrospective population‐based cohort study identifying target areas for prevention of acute lower respiratory infections in children. BMC Public Health. 2010;10(1):757. 21138593 10.1186/1471-2458-10-757 PMC3004840

[10] Marsden‐Haug N , Foster VB , Gould PL , Elbert E , Wang H , Pavlin JA . Code‐based syndromic surveillance for influenza like illness by international classification of diseases Ninth Revision. Emerg Infect Dis. 2007;13(2):207‐216. 17479881 10.3201/eid1302.060557 PMC2725845

[11] Zheng W , Aitken R , Muscatello DJ , Churches T . Potential for early warning of viral influenza activity in the community by monitoring clinical diagnoses of influenza in hospital emergency departments. BMC Public Health. 2007;19(7):250. 10.1186/1471-2458-7-250 PMC2075512 17877836

[12] Elliot A . Syndromic surveillance: the next phase of public health monitoring during the H1N1 influenza pandemic? Euro Surveill. 2009; 14(44):19391. 19941780

[13] Robert Koch Institute . Report on the epidemiology of influenza in Germany season 2016/17. German Working Group Influenza. 2017; https://influenza.rki.de/Saisonberichte/2016.pdf.

[14] Köpke K , Prahm K , Buda S , Haas W . Evaluation of an ICD‐10‐based electronic surveillance of acute respiratory infections (SEED^ARE^) in Germany. Bundesgesundheitsblatt Gesundheitsforschung Gesundheitsschutz. 2016;59(11):1484‐1491. 27738704 10.1007/s00103-016-2454-0

[15] Buda S , Tolksdorf K , Schuler E , Kuhlen R , Haas W . Establishing an ICD‐10 code based SARI‐surveillance in Germany ‐ description of the system and first results from five recent influenza seasons. BMC Public Health. 2017;17(1):612. 28666433 10.1186/s12889-017-4515-1 PMC5493063

[16] Feemster KA , Leckerman KH , Middleton M , et al. Use of administrative data for the identification of laboratory‐confirmed influenza infection: the validity ofinfluenza‐specific ICD‐9 codes. J Pediatric Infect Dis Soc. 2013;2(1):63‐66. 26619444 10.1093/jpids/pis052

[17] Amodio E , Tramuto F , Costantino C , et al. Diagnosis of influenza: only a problem of coding? Med Princ Pract. 2014;23(6):568‐573. 25059566 10.1159/000364780 PMC5586933

[18] Moore HC , Lehmann D , de Klerk N , et al. How accurate are international classification of diseases‐10 diagnosis codes in detecting influenza and pertussis hospitalizations in children? J Pediatric Infect Dis Soc. 2014;3(3):255‐260. 26625389 10.1093/jpids/pit036

[19] Pisesky A , Benchimol EI , Wong CA , et al. Incidence of hospitalization for respiratory syncytial virus infection amongst children in ontario, canada: a population‐based study using validated health administrative data. PLoS ONE. 2016; 11(3):e0150416. 26958849 10.1371/journal.pone.0150416 PMC4784925

[20] Bloom‐Feshbach K , Alonso WJ , Charu V , et al. Latitudinal variations in seasonal activity of influenza and respiratory syncytial virus (RSV): a global comparative review. PLoS ONE. 2013;8(2):e54445. 23457451 10.1371/journal.pone.0054445 PMC3573019

[21] Broberg EK , Waris M , Johansen K , Snacken R , Penttinen P . European Influenza Surveillance Network . Seasonality and geographical spread of respiratory syncytial virus epidemics in 15 European countries, 2010 to 2016. Euro Surveill. 2018;23(5), 17-00284. 10.2807/1560-7917.ES.2018.23.5.17-00284 PMC5801642 29409569

[22] Gross M , Brune T , Jorch G , Rabe H , Hentschel R . Significance of respiratory syncytial virus (RSV) infection in the 1st year of life. Infection. 2000;28(1):34‐37. 10697789 10.1007/s150100050008

[23] Nagayama Y , Tsubaki T , Sawada K , Taguchi K , Nakayama S , Toba T . Age and sex as factors of response to RSV infections among those with previous history of wheezing. Pediatr Allergy Immunol. 2006;17(5):376‐381. 16846457 10.1111/j.1399-3038.2006.00404.x

[24] Florin TA , Plint AC , Zorc JJ . Viral bronchiolitis. Lancet. 2017;389(10065):211‐224. 27549684 10.1016/S0140-6736(16)30951-5 PMC6765220

[25] Tannhof C . Acute bronchitis. DoctorConsult. 2011;2:e213‐e215.

[26] Rose EB , Wheatley A , Langley G , Gerber S , Haynes A . Respiratory syncytial virus seasonality ‐ United States, 2014–2017. MMWR Morb Mortal Wkly Rep. 2018;67(2):71‐76. 29346336 10.15585/mmwr.mm6702a4 PMC5772804

[27] Janet S , Broad J , Snape MD . Respiratory syncytial virus seasonality and its implications on prevention strategies. Hum Vaccin Immunother. 2018;14(1):234‐244. 29194014 10.1080/21645515.2017.1403707 PMC5791579

[28] Weigl JA , Puppe W , Belke O , Neusüss J , Bagci F , Schmitt HJ . The descriptive epidemiology of severe lower respiratory tract infections in children in Kiel, Germany. Klin Padiatr. 2005;217(5):259‐267. 16167272 10.1055/s-2004-820352

[29] Falsey AR , Walsh EE . Respiratory syncytial virus infection in elderly adults. Drugs Aging. 2005;22(7):577‐587. 16038573 10.2165/00002512-200522070-00004 PMC7099998

[30] Malosh RE , Martin ET , Callear AP , et al. Respiratory syncytial virus hospitalization in middle‐aged and older adults. J Clin Virol. 2017;96:37‐43. 28942341 10.1016/j.jcv.2017.09.001 PMC5889293

