# Supplementary material for: Evaluation of using ICD‐10 code data for respiratory syncytial virus surveillance
Source: Influenza Other Respir Viruses. 2019 Jun 17;14(6):630–7. doi: 10.1111/irv.12665 (PMC7578302; doi:10.1111/irv.12665)
Supplement: Supplementary file 1 [file IRV-14-630-s001.docx]

**Appendix**

**Description of the ICD-10-based digital surveillance systems and virological surveillance for influenza and other acute respiratory infections (ARI) at the Robert Koch Institute, Germany**

|  | **Surveillance participants** | **Region of participants** | **Data collection** | **Collected data** | **Total number of detection** |
| --- | --- | --- | --- | --- | --- |
| ***Primary care*** |  |  |  |  |  |
| **Sentinel electronic data collection system for ARI based on ICD-10 codes (SEED^ARE^)** | 193 practices:   - 107 general practices; - 46 pediatric practices; - 26 internist practices; - 14 practices with different specialties | 16 federal states | Digital data of medical consultations with any of ARI ICD-10 code diagnoses (J00-J22, J44.0, B34.9),  once a week | Age, gender, region, ICD-10 code diagnosis, consultation date, information on inability to work, hospitalization, influenza vaccination status | 1,087,243 ARI consultations, week 40/2007-13/2017 |
| **Virological surveillance** | 222 practices | 16 federal states | Pediatric practices:  3 respiratory specimens of patients with ARI or influenza like illness (ILI) per practice,  once a week;  General and internist practices:  5 respiratory specimens of patients with ARI or ILI per practice,  once a week | Age, gender, region, sampling date, symptoms, laboratory findings | 23,834 respiratory specimens tested for RSV, week 40/2010-18/2017 |
|  |  |  |  |  |  |
| ***Secondary care*** |  |  |  |  |  |
| **ICD-10 based hospital surveillance for severe acute respiratory infections (ICOSARI)** | 84 hospitals | 13 federal states | Digital data of hospitalizations with any of respiratory ICD-10 code diagnoses (chapter X: J00-J99) as primary or secondary discharge diagnosis,  weekly updated | Age, gender, region, primary and secondary discharge ICD-10 code diagnosis, admission diagnosis, admission date, discharge date, duration of stay in hospital and in intensive care unit, duration of ventilatory support, outcome | 1,417,700 respiratory disease hospitalizations,  week 01/2009-15/2017 |
